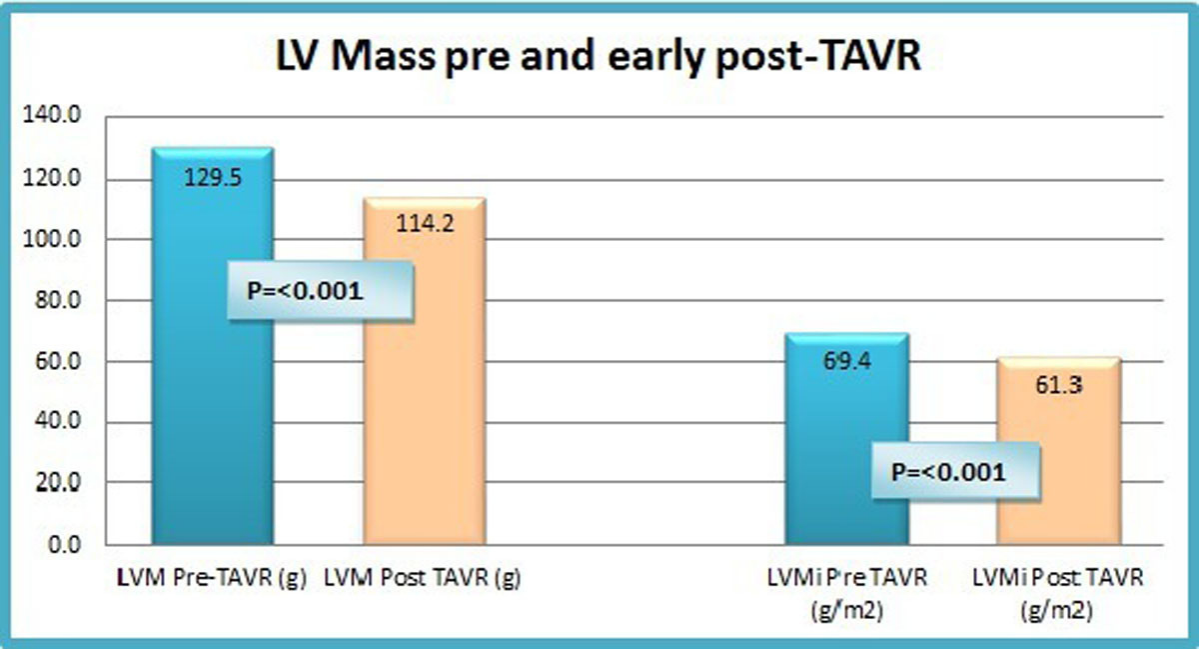# Left ventricular mass regression may occur very early following transcatheter aortic valve implantation for severe aortic stenosis

**DOI:** 10.1186/1532-429X-17-S1-P341

**Published:** 2015-02-03

**Authors:** Laura E Dobson, Tarique A Musa, Timothy A Fairbairn, Akhlaque Uddin, Daniel J Blackman, David P Ripley, Peter P Swoboda, Adam K McDiarmid, Bara Erhayiem, Pankaj Garg, Sven Plein, John P Greenwood

**Affiliations:** 1 Yorkshire Heart Centre, Leeds Teaching Hospitals Trust, Leeds, UK; 2 Cardiac MRI, Multidisciplinary Cardiovascular Research Centre & Leeds institute of Cardiovascular and Metabolic Medicine, Leeds, UK

## Background

The effect of transcatheter aortic valve implantation (TAVR) on left ventricular remodelling early following valve implantation has not previously been evaluated using cardiac magnetic resonance (CMR). Early left ventricular mass (LVM) regression (assessed using echocardiography) has been linked to a 50% reduction in re-hospitalisation in the first year following TAVR. We sought to establish using CMR, the reference standard non-invasive imaging technique for LVM quantification, whether LVM regression occurs early post-TAVR.

## Methods

27 patients with symptomatic severe aortic stenosis undergoing TAVR were prospectively enrolled between June 2013 and April 2014. Patients with contraindications to CMR were excluded and all patients provided informed written consent. All patients underwent an identical 1.5T CMR protocol (Intera, Philips) prior to and at a median of 5 days following TAVR (IQR 2 days). Multislice, multi-phase cine imaging was carried out using a standard steady-state free procession pulse sequence in the short axis to cover the entire left ventricle. Quantitative analysis was performed using dedicated computer software (CVI^42^, Circle Cardiovascular Imaging, Alberta, Canada). LVM was calculated by the formula (epicardial volume - endocardial volume) x myocardial density (1.05g/cm^3^).

## Results

Basic patient, echocardiographic and procedural characteristics can be seen in Table 1.

**Table 1:** Patient, echocardiographic and procedural characteristics. Mean ± SD.

Age, y	78 ± 6
Gender, (male %)	19 (70%)

STS Mortality score, %	4.1 ± 3

STS Morbidity/mortality score, %	21.6 ± 7

Hypertension (%)	12 (44)

NYHA Grade	2.9 ± 0.5

AVA (cm2)	0.62 ± 0.21

Peak pressure drop (mmHg)	90 ± 27

TAVI Type	

Medtronic Corevalve (%)	14 (52)

Boston Lotus (%)	12 (44)

Medtronic Engager (%)	1 (4)

At a median of 5 days following TAVR, mean LVM regressed by approximately 12% from 129.5± 32.5g to 114.2±31g and indexed LVM (LVMi) reduced from 69.4± 15.2g/m^2^ to 61.3±15.1g/m^2^ (Graph 1). There was no significant change in indexed left ventricular diastolic volume (96.9 ± 24.5 to 96.8 ± 19.3ml/m2, p=0.79), LV ejection fraction (54.0± 14.6 to 58.4 ± 27.4%, p=0.49) or indexed left atrial volume (71.5±22.6 to 67.7±22.1ml/m2, p=0.43). LVM regression was similar between the Medtronic and Boston valve types (14.3±10.4g Vs 16.5 ± 9.9g, p=0.58). There was no correlation between Valvulo-arterial impedance (ZVA) and LVM regression (r=0.02, p=0.93). There was no difference in mean LVMi regression according to gender (women 8.3±2.8g/m^2^ Vs men 8.0±5.7g/m^2^, p=0.83). TAVR valve size did not appear to impact on early LVM regression. LVMi regression was similar between those with a normal baseline LVM and those with an elevated baseline LVM (defined as 83g/m^2^ in men and 67g/m^2^) in women (7.8±4.8g Vs 9.0±5.7g, p= 0.60).

16 patients had evidence of late gadolinium enhancement (LGE) at baseline. There was a trend towards increased LVMi regression in those without LGE at baseline compared with those with LGE (10.1±4.7g/m^2^ Vs 6.4±4.7g/m^2^, p= 0.07).

## Conclusions

LVM regression begins very early following TAVR and occurs before changes in cavity size or ejection fraction which is likely to be a result of an immediate reduction in wall stress. The type of valve implanted does not appear to influence early LVM regression.

## Funding

This study was part funded by the British Heart Foundation (PG/11/126/29321).

**Figure 1:**